# Supplementary material for: Characterising the influence of cerebellum on the neuroplastic modulation of intracortical motor circuits
Source: PLoS One. 2020 Jul 10;15(7):e0236005. doi: 10.1371/journal.pone.0236005 (PMC7351163; doi:10.1371/journal.pone.0236005)
Supplement: Attachment — Submitted filename: Response to reviewers.docx [file pone.0236005.s002.docx]

*I have one minor comment with regard to sham stimulation - depending on the intensities used for SICF/ITMS, paired pulse ISIs could be facilitatory, rather than inhibitory. I think this point might be illustrated in the figures of Ilic and colleagues (2002). E.g. I think it is even possible to have facilitation at ISI 2ms, depending on the intensity. Personally I would probably avoid including ISI 1.5ms (or any in the range of 1.1-1.7ms) in the sham condition and try to select ISIs that are intermediate to peaks and troughs - ISIs that would be anticipated to show little facilitation or inhibition at the intensities employed. Ultimately, there is probably no ideal way to circumvent this issue but I raise it for the author's consideration.*

Response:

Thanks to the reviewer for their additional comment. As suggested, the issue of sham stimulation for the iTMS protocol represents a significant challenge. However, we appreciate the point being made, and will therefore modify the sham protocol to only use ISI’s that represents transitions between peaks and troughs of facilitation. Consequently, the sham protocol will include equal repetitions of ISI’s 1.8, 2.3, 3.3, 3.8 and 4.7 ms. These intervals were identified based on facilitation curves presented by several previous studies (Ziemann *et al.*, 1998; Delvendahl *et al.*, 2014; Cirillo *et al.*, 2015; Opie *et al.*, 2018). The revised manuscript has been updated to include these details (page 8).

Cirillo J, Calabro FJ & Perez MA. (2015). Impaired organization of paired-pulse TMS-induced I-waves after human spinal cord injury. *Cereb Cortex* **26,** 2167-2177.

Delvendahl I, Lindemann H, Jung NH, Pechmann A, Siebner HR & Mall V. (2014). Influence of waveform and current direction on short-interval intracortical facilitation: a paired-pulse TMS study. *Brain Stimul* **7,** 49-58.

Opie GM, Cirillo J & Semmler JG. (2018). Age‐related changes in late I‐waves influence motor cortex plasticity induction in older adults. *J Physiol* **596,** 2597-2609.

Ziemann U, Tergau F, Wassermann EM, Wischer S, Hildebrandt J & Paulus W. (1998). Demonstration of facilitatory I wave interaction in the human motor cortex by paired transcranial magnetic stimulation. *J Physiol* **511,** 181-190.
